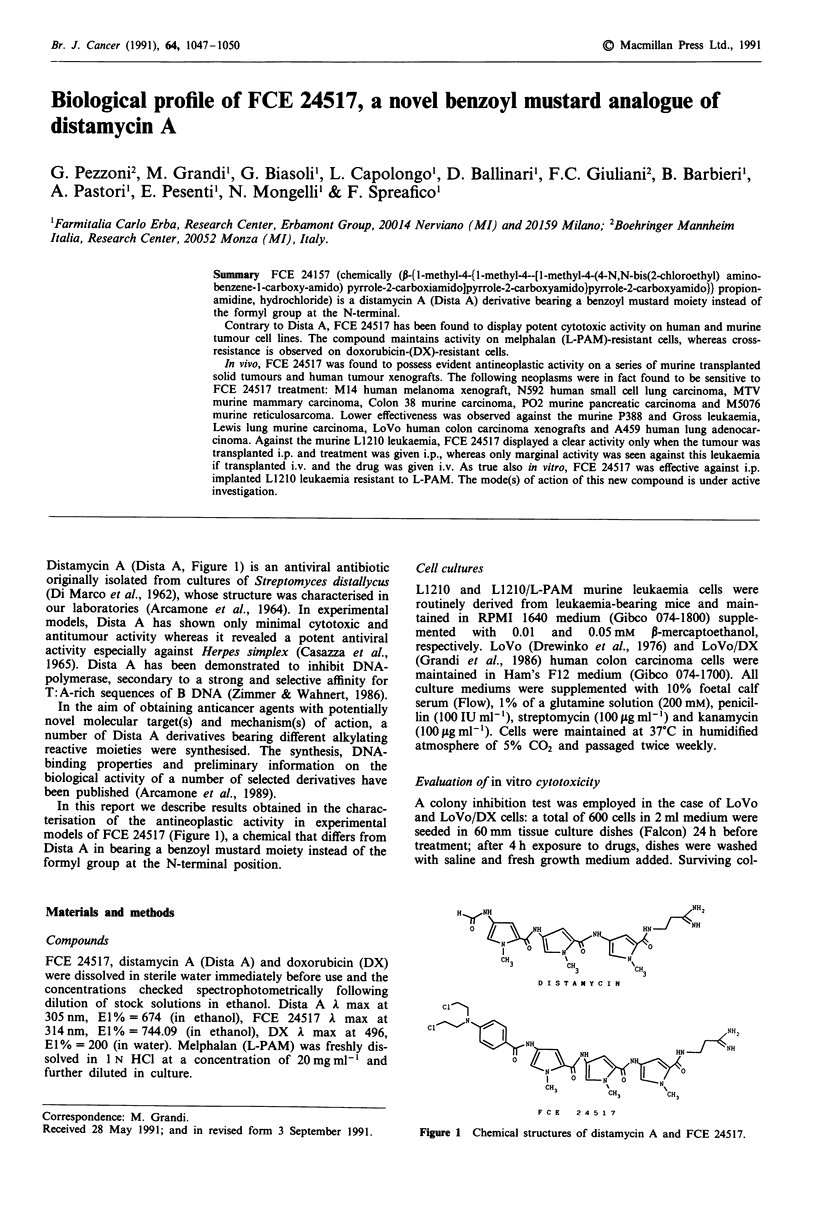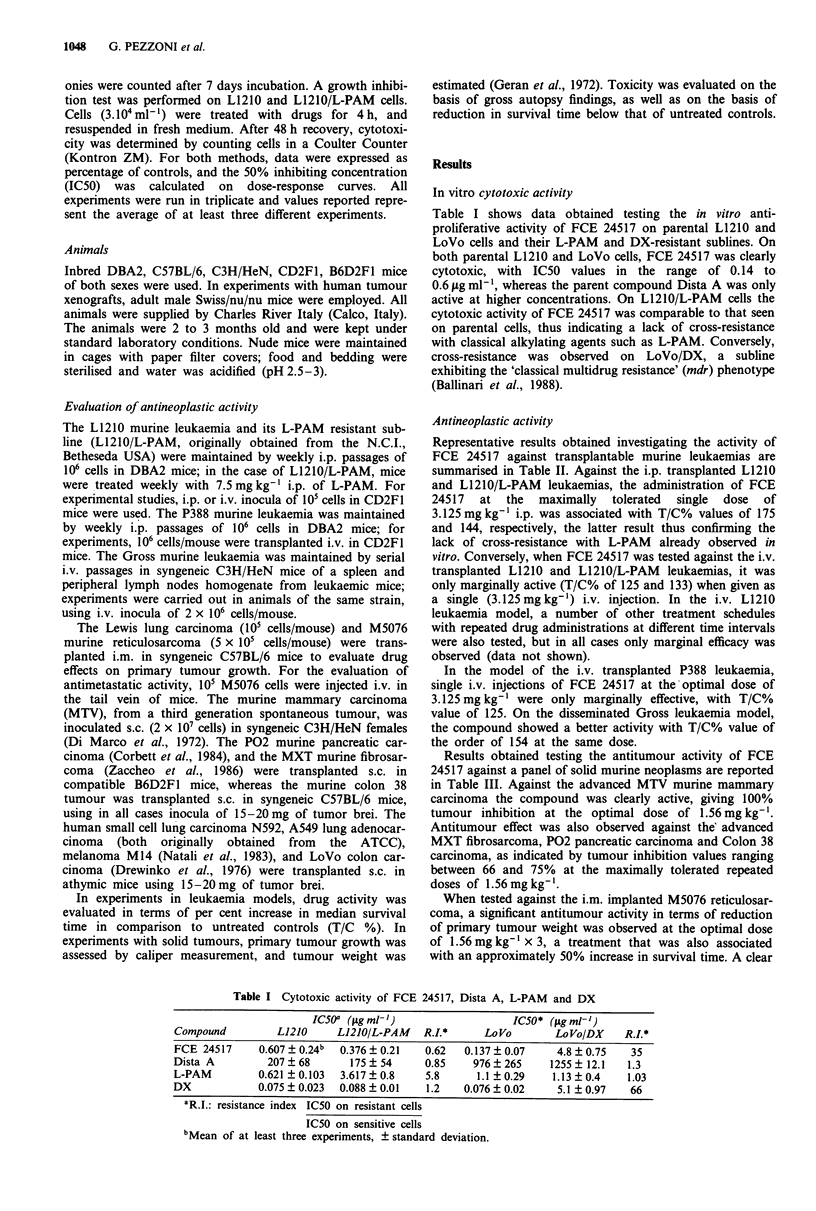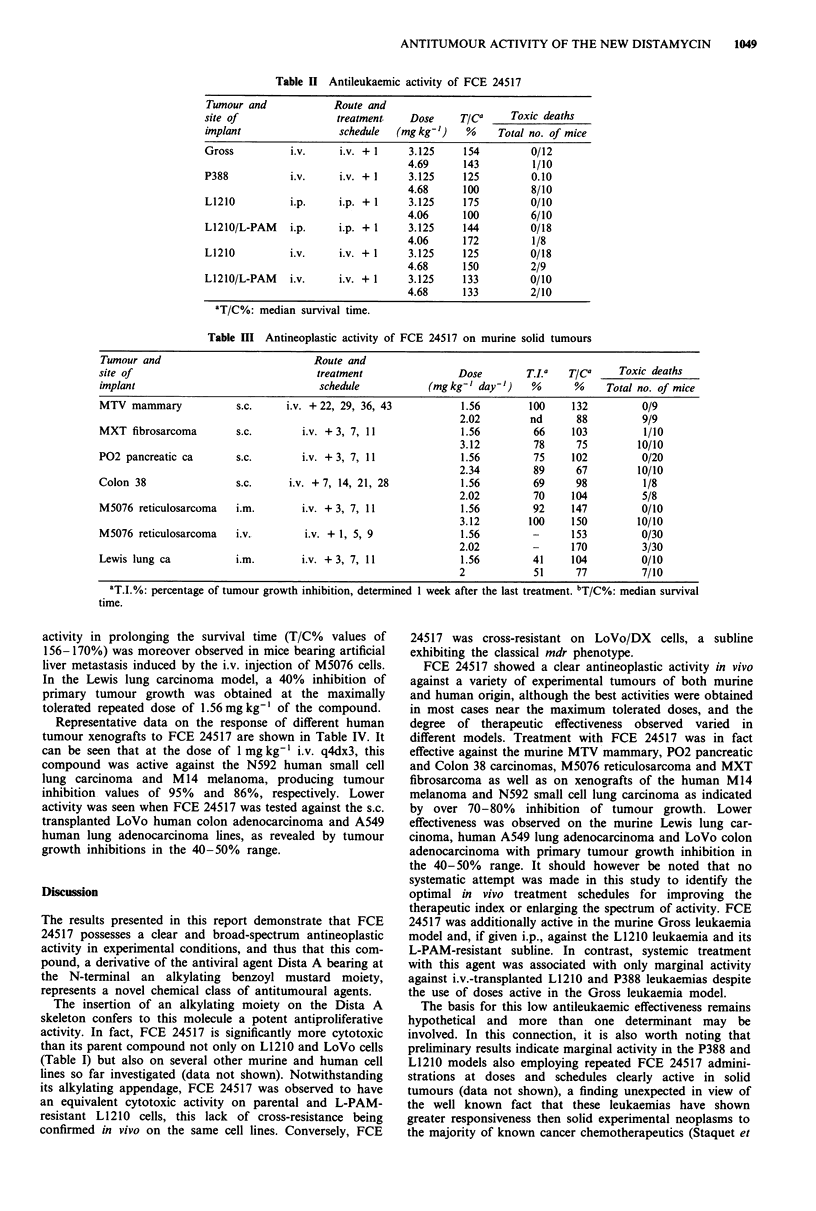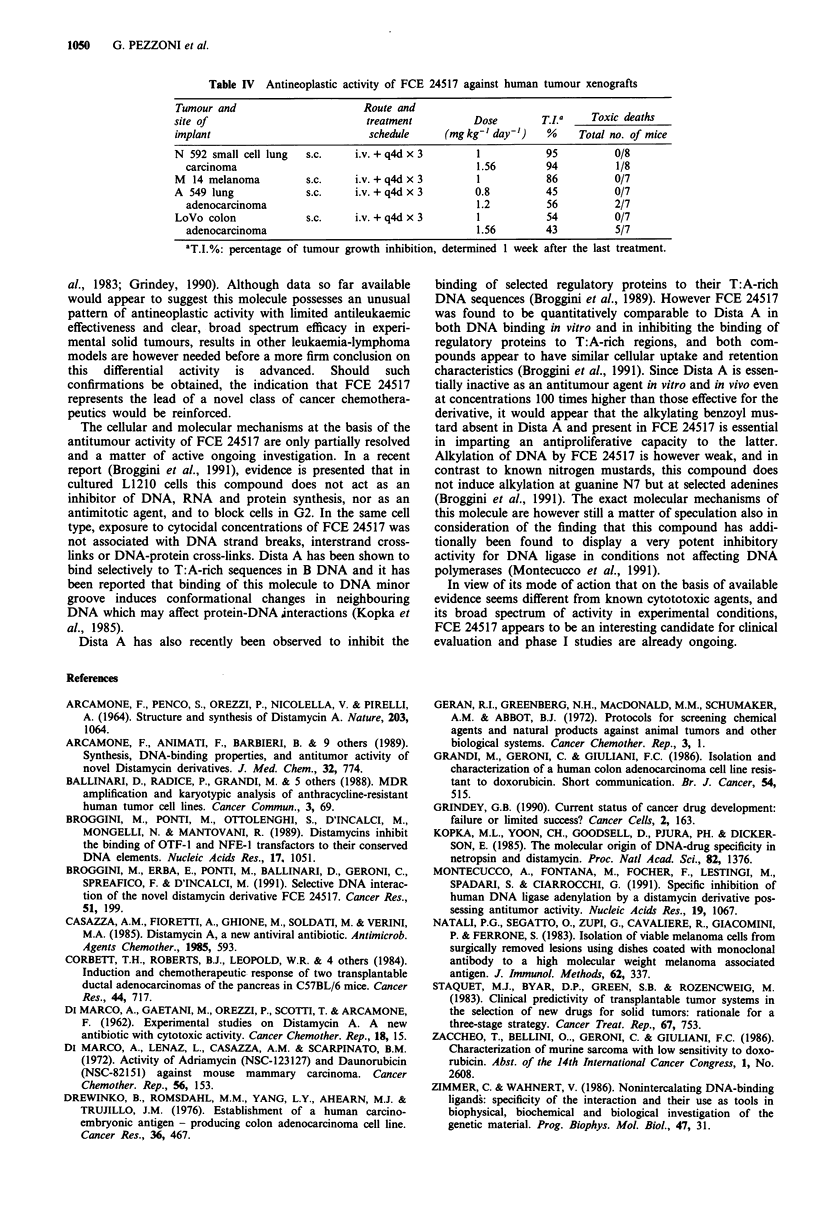# Biological profile of FCE 24517, a novel benzoyl mustard analogue of distamycin A.

**DOI:** 10.1038/bjc.1991.463

**Published:** 1991-12

**Authors:** G. Pezzoni, M. Grandi, G. Biasoli, L. Capolongo, D. Ballinari, F. C. Giuliani, B. Barbieri, A. Pastori, E. Pesenti, N. Mongelli

**Affiliations:** Farmitalia Carlo Erba, Research Center, Erbamont Group, Milano, Italy.

## Abstract

FCE 24157 (chemically (beta-[1-methyl-4-(1-methyl-4--[1-methyl-4-(4-N,N- bis(2-chloroethyl) amino-benzene-1-carboxy-amido) pyrrole-2-carboxiamido]pyrrole-2-carboxyamido)pyrrole-2-c arboxyamido]) propionamidine, hydrochloride) is a distamycin A (Dista A) derivative bearing a benzoyl mustard moiety instead of the formyl group at the N-terminal. Contrary to Dista A, FCE 24517 has been found to display potent cytotoxic activity on human and murine tumour cell lines. The compound maintains activity on melphalan (L-PAM)-resistant cells, whereas cross-resistance is observed on doxorubicin-(DX)-resistant cells. In vivo, FCE 24517 was found to possess evident antineoplastic activity on a series of murine transplanted solid tumours and human tumour xenografts. The following neoplasms were in fact found to be sensitive to FCE 24517 treatment: M14 human melanoma xenograft, N592 human small cell lung carcinoma, MTV murine mammary carcinoma, Colon 38 murine carcinoma, PO2 murine pancreatic carcinoma and M5076 murine reticulosarcoma. Lower effectiveness was observed against the murine P388 and Gross leukaemia, Lewis lung murine carcinoma, LoVo human colon carcinoma xenografts and A459 human lung adenocarcinoma. Against the murine L1210 leukaemia, FCE 24517 displayed a clear activity only when the tumour was transplanted i.p. and treatment was given i.p., whereas only marginal activity was seen against this leukaemia if transplanted i.v. and the drug was given i.v. As true also in vitro, FCE 24517 was effective against i.p. implanted L1210 leukaemia resistant to L-PAM. The mode(s) of action of this new compound is under active investigation.


Br. J. Cancer (1991), 64, 1047–1050 © Macmillan Press Ltd., 1991

### Biological profile of FCE 24517, a novel benzoyl mustard analogue of distamycin A

G. Pezzoni[2], M. Grandi[1], G. Biasoli[1], L. Capolongo[1], D. Ballinari[1], F.C. Giuliani[2], B. Barbieri[1], A. Pastori[1], E. Pesenti[1], N. Mongelli[1] & F. Spreafico[1]

[1]*Farmitalia Carlo Erba, Research Center, Erbamont Group, 20014 Nerviano (MI) and 20159 Milano;* [2]*Boehringer Mannheim Italia, Research Center, 20052 Monza (MI), Italy.*

**Summary** FCE 24157 (chemically (β-{1-methyl-4-{1-methyl-4--[1-methyl-4-(4-N,N-bis(2-chloroethyl) amino-benzene-1-carboxy-amido) pyrrole-2-carboxiamido]pyrrole-2-carboxyamido}pyrrole-2-carboxyamido}} propion-amidine, hydrochloride) is a distamycin A (Dista A) derivative bearing a benzoyl mustard moiety instead of the formyl group at the N-terminal.

Contrary to Dista A, FCE 24517 has been found to display potent cytotoxic activity on human and murine tumour cell lines. The compound maintains activity on melphalan (L-PAM)-resistant cells, whereas cross-resistance is observed on doxorubicin-(DX)-resistant cells.

*In vivo*, FCE 24517 was found to possess evident antineoplastic activity on a series of murine transplanted solid tumours and human tumour xenografts. The following neoplasms were in fact found to be sensitive to FCE 24517 treatment: M14 human melanoma xenograft, N592 human small cell lung carcinoma, MTV murine mammary carcinoma, Colon 38 murine carcinoma, PO2 murine pancreatic carcinoma and M5076 murine reticulosarcoma. Lower effectiveness was observed against the murine P388 and Gross leukaemia, Lewis lung murine carcinoma, LoVo human colon carcinoma xenografts and A459 human lung adenocarcinoma. Against the murine L1210 leukaemia, FCE 24517 displayed a clear activity only when the tumour was transplanted i.p. and treatment was given i.p., whereas only marginal activity was seen against this leukaemia if transplanted i.v. and the drug was given i.v. As true also *in vitro*, FCE 24517 was effective against i.p. implanted L1210 leukaemia resistant to L-PAM. The mode(s) of action of this new compound is under active investigation.

Distamycin A (Dista A, Figure 1) is an antiviral antibiotic originally isolated from cultures of *Streptomyces distallycus* (Di Marco *et al.*, 1962), whose structure was characterised in our laboratories (Arcamone *et al.*, 1964). In experimental models, Dista A has shown only minimal cytotoxic and antitumour activity whereas it revealed a potent antiviral activity especially against *Herpes simplex* (Casazza *et al.*, 1965). Dista A has been demonstrated to inhibit DNA-polymerase, secondary to a strong and selective affinity for T:A-rich sequences of B DNA (Zimmer & Wahnert, 1986).

In the aim of obtaining anticancer agents with potentially novel molecular target(s) and mechanism(s) of action, a number of Dista A derivatives bearing different alkylating reactive moieties were synthesised. The synthesis, DNA-binding properties and preliminary information on the biological activity of a number of selected derivatives have been published (Arcamone *et al.*, 1989).

In this report we describe results obtained in the characterisation of the antineoplastic activity in experimental models of FCE 24517 (Figure 1), a chemical that differs from Dista A in bearing a benzoyl mustard moiety instead of the formyl group at the N-terminal position.

### Materials and methods

### *Compounds*

FCE 24517, distamycin A (Dista A) and doxorubicin (DX) were dissolved in sterile water immediately before use and the concentrations checked spectrophotometrically following dilution of stock solutions in ethanol. Dista A λ max at 305 nm, E1% = 674 (in ethanol), FCE 24517 λ max at 314 nm, E1% = 744.09 (in ethanol), DX λ max at 496, E1% = 200 (in water). Melphalan (L-PAM) was freshly dissolved in 1 N HCl at a concentration of 20 mg $ml^{-1}$ and further diluted in culture.

Correspondence: M. Grandi.
Received 28 May 1991; and in revised form 3 September 1991.

### *Cell cultures*

L1210 and L1210/L-PAM murine leukaemia cells were routinely derived from leukaemia-bearing mice and maintained in RPMI 1640 medium (Gibco 074-1800) supplemented with 0.01 and 0.05 mM β-mercaptoethanol, respectively. LoVo (Drewinko *et al.*, 1976) and LoVo/DX (Grandi *et al.*, 1986) human colon carcinoma cells were maintained in Ham's F12 medium (Gibco 074-1700). All culture mediums were supplemented with 10% foetal calf serum (Flow), 1% of a glutamine solution (200 mM), penicillin (100 IU $ml^{-1}$), streptomycin (100 μg $ml^{-1}$) and kanamycin (100 μg $ml^{-1}$). Cells were maintained at 37°C in humidified atmosphere of 5% $CO_2$ and passaged twice weekly.

### *Evaluation of* in vitro *cytotoxicity*

A colony inhibition test was employed in the case of LoVo and LoVo/DX cells: a total of 600 cells in 2 ml medium were seeded in 60 mm tissue culture dishes (Falcon) 24 h before treatment; after 4 h exposure to drugs, dishes were washed with saline and fresh growth medium added. Surviving col-

**Figure 1** Chemical structures of distamycin A and FCE 24517.

onies were counted after 7 days incubation. A growth inhibition test was performed on L1210 and L1210/L-PAM cells. Cells ($3.10^4$ ml$^{-1}$) were treated with drugs for 4 h, and resuspended in fresh medium. After 48 h recovery, cytotoxicity was determined by counting cells in a Coulter Counter (Kontron ZM). For both methods, data were expressed as percentage of controls, and the 50% inhibiting concentration (IC50) was calculated on dose-response curves. All experiments were run in triplicate and values reported represent the average of at least three different experiments.

### *Animals*

Inbred DBA2, C57BL/6, C3H/HeN, CD2F1, B6D2F1 mice of both sexes were used. In experiments with human tumour xenografts, adult male Swiss/nu/nu mice were employed. All animals were supplied by Charles River Italy (Calco, Italy). The animals were 2 to 3 months old and were kept under standard laboratory conditions. Nude mice were maintained in cages with paper filter covers; food and bedding were sterilised and water was acidified (pH 2.5–3).

### *Evaluation of antineoplastic activity*

The L1210 murine leukaemia and its L-PAM resistant subline (L1210/L-PAM, originally obtained from the N.C.I., Betheseda USA) were maintained by weekly i.p. passages of $10^6$ cells in DBA2 mice; in the case of L1210/L-PAM, mice were treated weekly with 7.5 mg kg$^{-1}$ i.p. of L-PAM. For experimental studies, i.p. or i.v. inocula of $10^5$ cells in CD2F1 mice were used. The P388 murine leukaemia was maintained by weekly i.p. passages of $10^6$ cells in DBA2 mice; for experiments, $10^6$ cells/mouse were transplanted i.v. in CD2F1 mice. The Gross murine leukaemia was maintained by serial i.v. passages in syngeneic C3H/HeN mice of a spleen and peripheral lymph nodes homogenate from leukaemic mice; experiments were carried out in animals of the same strain, using i.v. inocula of $2 \times 10^6$ cells/mouse.

The Lewis lung carcinoma ($10^5$ cells/mouse) and M5076 murine reticulosarcoma ($5 \times 10^5$ cells/mouse) were transplanted i.m. in syngeneic C57BL/6 mice to evaluate drug effects on primary tumour growth. For the evaluation of antimetastatic activity, $10^5$ M5076 cells were injected i.v. in the tail vein of mice. The murine mammary carcinoma (MTV), from a third generation spontaneous tumour, was inoculated s.c. ($2 \times 10^7$ cells) in syngeneic C3H/HeN females (Di Marco *et al.*, 1972). The PO2 murine pancreatic carcinoma (Corbett *et al.*, 1984), and the MXT murine fibrosarcoma (Zaccheo *et al.*, 1986) were transplanted s.c. in compatible B6D2F1 mice, whereas the murine colon 38 tumour was transplanted s.c. in syngeneic C57BL/6 mice, using in all cases inocula of 15–20 mg of tumor brei. The human small cell lung carcinoma N592, A549 lung adenocarcinoma (both originally obtained from the ATCC), melanoma M14 (Natali *et al.*, 1983), and LoVo colon carcinoma (Drewinko *et al.*, 1976) were transplanted s.c. in athymic mice using 15–20 mg of tumor brei.

In experiments in leukaemia models, drug activity was evaluated in terms of per cent increase in median survival time in comparison to untreated controls (T/C %). In experiments with solid tumours, primary tumour growth was assessed by caliper measurement, and tumour weight was estimated (Geran *et al.*, 1972). Toxicity was evaluated on the basis of gross autopsy findings, as well as on the basis of reduction in survival time below that of untreated controls.

### Results

### In vitro *cytotoxic activity*

Table I shows data obtained testing the *in vitro* antiproliferative activity of FCE 24517 on parental L1210 and LoVo cells and their L-PAM and DX-resistant sublines. On both parental L1210 and LoVo cells, FCE 24517 was clearly cytotoxic, with IC50 values in the range of 0.14 to 0.6 μg ml$^{-1}$, whereas the parent compound Dista A was only active at higher concentrations. On L1210/L-PAM cells the cytotoxic activity of FCE 24517 was comparable to that seen on parental cells, thus indicating a lack of cross-resistance with classical alkylating agents such as L-PAM. Conversely, cross-resistance was observed on LoVo/DX, a subline exhibiting the 'classical multidrug resistance' (*mdr*) phenotype (Ballinari *et al.*, 1988).

### *Antineoplastic activity*

Representative results obtained investigating the activity of FCE 24517 against transplantable murine leukaemias are summarised in Table II. Against the i.p. transplanted L1210 and L1210/L-PAM leukaemias, the administration of FCE 24517 at the maximally tolerated single dose of 3.125 mg kg$^{-1}$ i.p. was associated with T/C% values of 175 and 144, respectively, the latter result thus confirming the lack of cross-resistance with L-PAM already observed *in vitro*. Conversely, when FCE 24517 was tested against the i.v. transplanted L1210 and L1210/L-PAM leukaemias, it was only marginally active (T/C% of 125 and 133) when given as a single (3.125 mg kg$^{-1}$) i.v. injection. In the i.v. L1210 leukaemia model, a number of other treatment schedules with repeated drug administrations at different time intervals were also tested, but in all cases only marginal efficacy was observed (data not shown).

In the model of the i.v. transplanted P388 leukaemia, single i.v. injections of FCE 24517 at the optimal dose of 3.125 mg kg$^{-1}$ were only marginally effective, with T/C% value of 125. On the disseminated Gross leukaemia model, the compound showed a better activity with T/C% value of the order of 154 at the same dose.

Results obtained testing the antitumour activity of FCE 24517 against a panel of solid murine neoplasms are reported in Table III. Against the advanced MTV murine mammary carcinoma the compound was clearly active, giving 100% tumour inhibition at the optimal dose of 1.56 mg kg$^{-1}$. Antitumour effect was also observed against the advanced MXT fibrosarcoma, PO2 pancreatic carcinoma and Colon 38 carcinoma, as indicated by tumour inhibition values ranging between 66 and 75% at the maximally tolerated repeated doses of 1.56 mg kg$^{-1}$.

When tested against the i.m. implanted M5076 reticulosarcoma, a significant antitumour activity in terms of reduction of primary tumour weight was observed at the optimal dose of 1.56 mg kg$^{-1}$ × 3, a treatment that was also associated with an approximately 50% increase in survival time. A clear

**Table I** Cytotoxic activity of FCE 24517, Dista A, L-PAM and DX

| Compound | *IC50[a] (μg ml⁻¹)* L1210 | L1210/L-PAM | R.I.* | *IC50* (μg ml⁻¹)* LoVo | LoVo/DX | R.I.* |
|---|---|---|---|---|---|---|
| FCE 24517 | 0.607 ± 0.24[b] | 0.376 ± 0.21 | 0.62 | 0.137 ± 0.07 | 4.8 ± 0.75 | 35 |
| Dista A | 207 ± 68 | 175 ± 54 | 0.85 | 976 ± 265 | 1255 ± 12.1 | 1.3 |
| L-PAM | 0.621 ± 0.103 | 3.617 ± 0.8 | 5.8 | 1.1 ± 0.29 | 1.13 ± 0.4 | 1.03 |
| DX | 0.075 ± 0.023 | 0.088 ± 0.01 | 1.2 | 0.076 ± 0.02 | 5.1 ± 0.97 | 66 |

[a]R.I.: resistance index $\frac{\text{IC50 on resistant cells}}{\text{IC50 on sensitive cells}}$

[b]Mean of at least three experiments, ± standard deviation.

**Table II** Antileukaemic activity of FCE 24517

| *Tumour and site of implant* | | *Route and treatment schedule* | *Dose (mg kg⁻¹)* | *T/C*[a] *%* | *Toxic deaths* *Total no. of mice* |
|---|---|---|---|---|---|
| Gross | i.v. | i.v. + 1 | 3.125 | 154 | 0/12 |
| | | | 4.69 | 143 | 1/10 |
| P388 | i.v. | i.v. + 1 | 3.125 | 125 | 0.10 |
| | | | 4.68 | 100 | 8/10 |
| L1210 | i.p. | i.p. + 1 | 3.125 | 175 | 0/10 |
| | | | 4.06 | 100 | 6/10 |
| L1210/L-PAM | i.p. | i.p. + 1 | 3.125 | 144 | 0/18 |
| | | | 4.06 | 172 | 1/8 |
| L1210 | i.v. | i.v. + 1 | 3.125 | 125 | 0/18 |
| | | | 4.68 | 150 | 2/9 |
| L1210/L-PAM | i.v. | i.v. + 1 | 3.125 | 133 | 0/10 |
| | | | 4.68 | 133 | 2/10 |

[a]T/C%: median survival time.

**Table III** Antineoplastic activity of FCE 24517 on murine solid tumours

| *Tumour and site of implant* | | *Route and treatment schedule* | *Dose ($mg\ kg^{-1}\ day^{-1}$)* | *T.I.*[a] *%* | *T/C*[a] *%* | *Toxic deaths* *Total no. of mice* |
|---|---|---|---|---|---|---|
| MTV mammary | s.c. | i.v. + 22, 29, 36, 43 | 1.56 | 100 | 132 | 0/9 |
| | | | 2.02 | nd | 88 | 9/9 |
| MXT fibrosarcoma | s.c. | i.v. + 3, 7, 11 | 1.56 | 66 | 103 | 1/10 |
| | | | 3.12 | 78 | 75 | 10/10 |
| PO2 pancreatic ca | s.c. | i.v. + 3, 7, 11 | 1.56 | 75 | 102 | 0/20 |
| | | | 2.34 | 89 | 67 | 10/10 |
| Colon 38 | s.c. | i.v. + 7, 14, 21, 28 | 1.56 | 69 | 98 | 1/8 |
| | | | 2.02 | 70 | 104 | 5/8 |
| M5076 reticulosarcoma | i.m. | i.v. + 3, 7, 11 | 1.56 | 92 | 147 | 0/10 |
| | | | 3.12 | 100 | 150 | 10/10 |
| M5076 reticulosarcoma | i.v. | i.v. + 1, 5, 9 | 1.56 | – | 153 | 0/30 |
| | | | 2.02 | – | 170 | 3/30 |
| Lewis lung ca | i.m. | i.v. + 3, 7, 11 | 1.56 | 41 | 104 | 0/10 |
| | | | 2 | 51 | 77 | 7/10 |

[a]T.I.%: percentage of tumour growth inhibition, determined 1 week after the last treatment. [b]T/C%: median survival time.

activity in prolonging the survival time (T/C% values of 156–170%) was moreover observed in mice bearing artificial liver metastasis induced by the i.v. injection of M5076 cells. In the Lewis lung carcinoma model, a 40% inhibition of primary tumour growth was obtained at the maximally tolerated repeated dose of $1.56\ mg\ kg^{-1}$ of the compound.

Representative data on the response of different human tumour xenografts to FCE 24517 are shown in Table IV. It can be seen that at the dose of $1\ mg\ kg^{-1}$ i.v. q4dx3, this compound was active against the N592 human small cell lung carcinoma and M14 melanoma, producing tumour inhibition values of 95% and 86%, respectively. Lower activity was seen when FCE 24517 was tested against the s.c. transplanted LoVo human colon adenocarcinoma and A549 human lung adenocarcinoma lines, as revealed by tumour growth inhibitions in the 40–50% range.

### Discussion

The results presented in this report demonstrate that FCE 24517 possesses a clear and broad-spectrum antineoplastic activity in experimental conditions, and thus that this compound, a derivative of the antiviral agent Dista A bearing at the N-terminal an alkylating benzoyl mustard moiety, represents a novel chemical class of antitumoural agents.

The insertion of an alkylating moiety on the Dista A skeleton confers to this molecule a potent antiproliferative activity. In fact, FCE 24517 is significantly more cytotoxic than its parent compound not only on L1210 and LoVo cells (Table I) but also on several other murine and human cell lines so far investigated (data not shown). Notwithstanding its alkylating appendage, FCE 24517 was observed to have an equivalent cytotoxic activity on parental and L-PAM-resistant L1210 cells, this lack of cross-resistance being confirmed *in vivo* on the same cell lines. Conversely, FCE 24517 was cross-resistant on LoVo/DX cells, a subline exhibiting the classical *mdr* phenotype.

FCE 24517 showed a clear antineoplastic activity *in vivo* against a variety of experimental tumours of both murine and human origin, although the best activities were obtained in most cases near the maximum tolerated doses, and the degree of therapeutic effectiveness observed varied in different models. Treatment with FCE 24517 was in fact effective against the murine MTV mammary, PO2 pancreatic and Colon 38 carcinomas, M5076 reticulosarcoma and MXT fibrosarcoma as well as on xenografts of the human M14 melanoma and N592 small cell lung carcinoma as indicated by over 70–80% inhibition of tumour growth. Lower effectiveness was observed on the murine Lewis lung carcinoma, human A549 lung adenocarcinoma and LoVo colon adenocarcinoma with primary tumour growth inhibition in the 40–50% range. It should however be noted that no systematic attempt was made in this study to identify the optimal *in vivo* treatment schedules for improving the therapeutic index or enlarging the spectrum of activity. FCE 24517 was additionally active in the murine Gross leukaemia model and, if given i.p., against the L1210 leukaemia and its L-PAM-resistant subline. In contrast, systemic treatment with this agent was associated with only marginal activity against i.v.-transplanted L1210 and P388 leukaemias despite the use of doses active in the Gross leukaemia model.

The basis for this low antileukaemic effectiveness remains hypothetical and more than one determinant may be involved. In this connection, it is also worth noting that preliminary results indicate marginal activity in the P388 and L1210 models also employing repeated FCE 24517 administrations at doses and schedules clearly active in solid tumours (data not shown), a finding unexpected in view of the well known fact that these leukaemias have shown greater responsiveness then solid experimental neoplasms to the majority of known cancer chemotherapeutics (Staquet *et*

**Table IV** Antineoplastic activity of FCE 24517 against human tumour xenografts

| *Tumour and site of implant* | | *Route and treatment schedule* | *Dose (mg $kg^{-1}$ $day^{-1}$)* | *T.I.*[a] *%* | *Toxic deaths* *Total no. of mice* |
|---|---|---|---|---|---|
| N 592 small cell lung | s.c. | i.v. + q4d × 3 | 1 | 95 | 0/8 |
| carcinoma | | | 1.56 | 94 | 1/8 |
| M 14 melanoma | s.c. | i.v. + q4d × 3 | 1 | 86 | 0/7 |
| A 549 lung | s.c. | i.v. + q4d × 3 | 0.8 | 45 | 0/7 |
| adenocarcinoma | | | 1.2 | 56 | 2/7 |
| LoVo colon | s.c. | i.v. + q4d × 3 | 1 | 54 | 0/7 |
| adenocarcinoma | | | 1.56 | 43 | 5/7 |

[a]T.I.%: percentage of tumour growth inhibition, determined 1 week after the last treatment.

al., 1983; Grindey, 1990). Although data so far available would appear to suggest this molecule possesses an unusual pattern of antineoplastic activity with limited antileukaemic effectiveness and clear, broad spectrum efficacy in experimental solid tumours, results in other leukaemia-lymphoma models are however needed before a more firm conclusion on this differential activity is advanced. Should such confirmations be obtained, the indication that FCE 24517 represents the lead of a novel class of cancer chemotherapeutics would be reinforced.

The cellular and molecular mechanisms at the basis of the antitumour activity of FCE 24517 are only partially resolved and a matter of active ongoing investigation. In a recent report (Broggini *et al.*, 1991), evidence is presented that in cultured L1210 cells this compound does not act as an inhibitor of DNA, RNA and protein synthesis, nor as an antimitotic agent, and to block cells in G2. In the same cell type, exposure to cytocidal concentrations of FCE 24517 was not associated with DNA strand breaks, interstrand cross-links or DNA-protein cross-links. Dista A has been shown to bind selectively to T:A-rich sequences in B DNA and it has been reported that binding of this molecule to DNA minor groove induces conformational changes in neighbouring DNA which may affect protein-DNA interactions (Kopka *et al.*, 1985).

Dista A has also recently been observed to inhibit the binding of selected regulatory proteins to their T:A-rich DNA sequences (Broggini *et al.*, 1989). However FCE 24517 was found to be quantitatively comparable to Dista A in both DNA binding *in vitro* and in inhibiting the binding of regulatory proteins to T:A-rich regions, and both compounds appear to have similar cellular uptake and retention characteristics (Broggini *et al.*, 1991). Since Dista A is essentially inactive as an antitumour agent *in vitro* and *in vivo* even at concentrations 100 times higher than those effective for the derivative, it would appear that the alkylating benzoyl mustard absent in Dista A and present in FCE 24517 is essential in imparting an antiproliferative capacity to the latter. Alkylation of DNA by FCE 24517 is however weak, and in contrast to known nitrogen mustards, this compound does not induce alkylation at guanine N7 but at selected adenines (Broggini *et al.*, 1991). The exact molecular mechanisms of this molecule are however still a matter of speculation also in consideration of the finding that this compound has additionally been found to display a very potent inhibitory activity for DNA ligase in conditions not affecting DNA polymerases (Montecucco *et al.*, 1991).

In view of its mode of action that on the basis of available evidence seems different from known cytototoxic agents, and its broad spectrum of activity in experimental conditions, FCE 24517 appears to be an interesting candidate for clinical evaluation and phase I studies are already ongoing.

### References

ARCAMONE, F., PENCO, S., OREZZI, P., NICOLELLA, V. & PIRELLI, A. (1964). Structure and synthesis of Distamycin A. *Nature*, **203**, 1064.

ARCAMONE, F., ANIMATI, F., BARBIERI, B. & 9 others (1989). Synthesis, DNA-binding properties, and antitumor activity of novel Distamycin derivatives. *J. Med. Chem.*, **32**, 774.

BALLINARI, D., RADICE, P., GRANDI, M. & 5 others (1988). MDR amplification and karyotypic analysis of anthracycline-resistant human tumor cell lines. *Cancer Commun.*, **3**, 69.

BROGGINI, M., PONTI, M., OTTOLENGHI, S., D'INCALCI, M., MONGELLI, N. & MANTOVANI, R. (1989). Distamycins inhibit the binding of OTF-1 and NFE-1 transfactors to their conserved DNA elements. *Nucleic Acids Res.*, **17**, 1051.

BROGGINI, M., ERBA, E., PONTI, M., BALLINARI, D., GERONI, C., SPREAFICO, F. & D'INCALCI, M. (1991). Selective DNA interaction of the novel distamycin derivative FCE 24517. *Cancer Res.*, **51**, 199.

CASAZZA, A.M., FIORETTI, A., GHIONE, M., SOLDATI, M. & VERINI, M.A. (1985). Distamycin A, a new antiviral antibiotic. *Antimicrob. Agents Chemother.*, **1985**, 593.

CORBETT, T.H., ROBERTS, B.J., LEOPOLD, W.R. & 4 others (1984). Induction and chemotherapeutic response of two transplantable ductal adenocarcinomas of the pancreas in C57BL/6 mice. *Cancer Res.*, **44**, 717.

DI MARCO, A., GAETANI, M., OREZZI, P., SCOTTI, T. & ARCAMONE, F. (1962). Experimental studies on Distamycin A. A new antibiotic with cytotoxic activity. *Cancer Chemother. Rep.*, **18**, 15.

DI MARCO, A., LENAZ, L., CASAZZA, A.M. & SCARPINATO, B.M. (1972). Activity of Adriamycin (NSC-123127) and Daunorubicin (NSC-82151) against mouse mammary carcinoma. *Cancer Chemother. Rep.*, **56**, 153.

DREWINKO, B., ROMSDAHL, M.M., YANG, L.Y., AHEARN, M.J. & TRUJILLO, J.M. (1976). Establishment of a human carcinoembryonic antigen – producing colon adenocarcinoma cell line. *Cancer Res.*, **36**, 467.

GERAN, R.I., GREENBERG, N.H., MACDONALD, M.M., SCHUMAKER, A.M. & ABBOT, B.J. (1972). Protocols for screening chemical agents and natural products against animal tumors and other biological systems. *Cancer Chemother. Rep.*, **3**, 1.

GRANDI, M., GERONI, C. & GIULIANI, F.C. (1986). Isolation and characterization of a human colon adenocarcinoma cell line resistant to doxorubicin. Short communication. *Br. J. Cancer*, **54**, 515.

GRINDEY, G.B. (1990). Current status of cancer drug development: failure or limited success? *Cancer Cells*, **2**, 163.

KOPKA, M.L., YOON, CH., GOODSELL, D., PJURA, PH. & DICKERSON, E. (1985). The molecular origin of DNA-drug specificity in netropsin and distamycin. *Proc. Natl Acad. Sci.*, **82**, 1376.

MONTECUCCO, A., FONTANA, M., FOCHER, F., LESTINGI, M., SPADARI, S. & CIARROCCHI, G. (1991). Specific inhibition of human DNA ligase adenylation by a distamycin derivative possessing antitumor activity. *Nucleic Acids Res.*, **19**, 1067.

NATALI, P.G., SEGATTO, O., ZUPI, G., CAVALIERE, R., GIACOMINI, P. & FERRONE, S. (1983). Isolation of viable melanoma cells from surgically removed lesions using dishes coated with monoclonal antibody to a high molecular weight melanoma associated antigen. *J. Immunol. Methods*, **62**, 337.

STAQUET, M.J., BYAR, D.P., GREEN, S.B. & ROZENCWEIG, M. (1983). Clinical predictivity of transplantable tumor systems in the selection of new drugs for solid tumors: rationale for a three-stage strategy. *Cancer Treat. Rep.*, **67**, 753.

ZACCHEO, T., BELLINI, O.., GERONI, C. & GIULIANI, F.C. (1986). Characterization of murine sarcoma with low sensitivity to doxorubicin. *Abst. of the 14th International Cancer Congress*, **1**, No. 2608.

ZIMMER, C. & WAHNERT, V. (1986). Nonintercalating DNA-binding ligands: specificity of the interaction and their use as tools in biophysical, biochemical and biological investigation of the genetic material. *Prog. Biophys. Mol. Biol.*, **47**, 31.